# Comparative Analysis of Full-Length Reference Gene Stability in *Phoebe zhennan* Under Primary Abiotic and Biotic Stresses

**DOI:** 10.3390/plants15111736

**Published:** 2026-06-03

**Authors:** Beibei Chen, Yingxuan Luo, Yuan Li, Zhenqi Liao, Zhongbiao Ding, Weiyi Liu

**Affiliations:** 1 Centre for Research in Biotechnology for Agriculture, Universiti Malaya, Kuala Lumpur 50603, Malaysia; 22050412@siswa.um.edu.my (B.C.); luozize@163.com (Y.L.); 2 Enshi Tujia and Miao Autonomous Prefecture Academy of Forestry, Enshi 445000, China; liyuan@esforestry.ac.cn (Y.L.); dingzb@esforestry.ac.cn (Z.D.); 3 School of Agriculture and Biotechnology, Sun Yat-sen University, Shenzhen 518107, China; liaozhq7@mail.sysu.edu.cn

**Keywords:** *Phoebe zhennan*, reference gene, RT-qPCR, expression stability, abiotic and biotic stress, transcriptomic normalization, *Colletotrichum fructicola*

## Abstract

(1) Reverse transcription quantitative real-time PCR (RT-qPCR) requires reliable reference genes for accurate data normalization; however, optimal reference genes for the economically and ecologically valuable timber species *Phoebe zhennan* remain uncharacterized; (2) Here, we selected nine candidate reference genes derived from full-length transcriptome sequencing to evaluate their expression stability across abiotic (drought) and biotic (*Colletotrichum fructicola* infection) stresses. Transcript abundance was analyzed via RT-qPCR using four distinct algorithms (Delta Ct, geNorm, NormFinder, and BestKeeper), with RefFinder used to reconcile analytical discrepancies and generate a definitive consensus ranking; (3) Our analysis showed that expression stability is highly context-dependent: *CYP20-1* and *HSP70-1* were the most stable reference genes under drought stress, whereas *Actin-101* and *Actin* constituted the optimal pair under disease stress. For cross-condition assessments, *Actin-101* and *β-Tubulin* served as the most reliable baseline combination. Subsequent empirical validation quantifying stress-responsive transcripts demonstrated a significant positive correlation between RT-qPCR relative expression and corresponding RNA-seq data (drought: *R* = 0.80; disease: *R* = 0.76); (4) This study identifies and validates the first set of reference genes for *P. zhennan*, providing a foundation for accurate gene expression analysis in this species, which is crucial for understanding its response to environmental stresses.

## 1. Introduction

Elucidating the molecular mechanisms underlying plant stress adaptation remains an important objective in plant biology. To precisely quantify these dynamic fluctuations in gene expression, reverse transcription–quantitative real-time polymerase chain reaction (RT-qPCR) serves as a widely used analytical tool, known for its sensitivity, broad dynamic range, and high-throughput reproducibility [1]. However, the analytical precision and biological validity of RT-qPCR data are entirely contingent upon the implementation of a rigorous data normalization strategy. To accurately correct for inherent experimental variations in initial RNA quantity, sample integrity, and reverse transcription efficiency, the expression profiles of target transcripts must be calibrated against highly stable internal reference genes [2].

Historically, researchers have defaulted to traditional housekeeping genes, such as *Actin*, glyceraldehyde-3-phosphate dehydrogenase (*GAPDH*), *18S ribosomal RNA (18S rRNA)*, *β-Tubulin* (*TUB*), and transcription elongation factor 1-alpha (*EF1α*), under the assumption that these basal metabolic transcripts maintain a constant expression equilibrium [3]. Yet, extensive transcriptomic evidence has challenged the existence of omnipotent, universally stable reference genes [4]; the expression stability of these widely used reference genes varies drastically depending on the specific tissues or experimental conditions [5,6]. For instance, the expression pattern of *18S rRNA* is highly unstable during the development of Chinese cabbage (*Brassica rapa*) flower buds [7] and under abiotic stress in moss (*Syntrichia caninervis*) [8]. In contrast, it remains remarkably consistent across diverse tissues and treatments in broomcorn millet (*Panicum miliaceum*) [9]. This condition-dependent stability is observed across various taxa. In soybean, *EF1A* and *ACT11* exhibit the highest stability under salt stress; however, *TUB4*, *TUA5*, and *EF1A* are optimal under drought conditions, while *EF1B* and *UKN2* are the most reliable during viral infection [10]. Similarly, in Chinese jujube (*Ziziphus jujuba*), *ACT1*, *His3*, and *PAIP* are the primary reference genes during fruit development. However, dark treatments require *ACT1*, *BTF3*, *GAPDH*, and *PAIP* [11]. Additionally, research in pea (*Pisum sativum*) has shown that traditional housekeeping genes, such as *18S rRNA*, *Actin*, and *β-tubulin*, exhibit significant expression variability under cold stress and *Sclerotinia* infection. In this context, *TIF* and *β-tubulin-3* have emerged as superior alternatives [12]. Ultimately, as demonstrated by Gutierrez et al. [13] through quantitative RT-PCR analysis across multiple tissues in *Arabidopsis thaliana* and hybrid aspen (*Populus tremula* × *P. tremuloides*), the inherent instability of many commonly used reference genes renders their unvalidated application as internal controls highly inappropriate. In accordance with the recently updated Minimum Information for Publication of Quantitative Real-Time PCR Experiments (MIQE) 2.0 guideline, it is strongly recommended to use the geometric mean of at least two validated, condition-specific reference genes to ensure robust data normalization [4,14]. Therefore, establishing the expression stability of reference genes specific to each plant species and experimental setup, rather than indiscriminately using references from model plants, has become an urgent necessity.

This methodological imperative is particularly critical for endangered, high-value forestry species such as *Phoebe zhennan*, a Category II national protected evergreen timber species of the Lauraceae family, renowned for yielding the highly prized “golden-thread” wood [15], which commands extraordinary market valuations ranging from approximately $750 per cubic meter for raw timber to over $8000 per ton for ancient, buried specimens [16,17]. The species is currently classified as vulnerable, facing severe demographic declines driven by historical habitat fragmentation and shifting climatic envelopes [18]. Furthermore, the survival and successful silvicultural establishment of *P. zhennan* seedlings are heavily threatened by localized extreme drought events induced by shifting climatic envelopes. Compounding this abiotic stress are recent, significant outbreaks of anthracnose and twig blight caused by the hemibiotrophic fungal pathogen *Colletotrichum fructicola* [19]. Recent field surveys have documented substantial disease incidence rates ranging from 22% to 30% in young *P. zhennan* plantations, establishing these dual factors as the major ecological threats currently driving seedling mortality. While macroscopic research into the physiological and biochemical resilience of *P. zhennan* is expanding [20], molecular investigations remain hindered by the lack of a systematically validated, species-specific RT-qPCR normalization framework. Currently, expression studies in non-model woody plants often utilize reference genes extrapolated from herbaceous models [13]. However, the complex, secondary metabolite-rich metabolomes of species like *P. zhennan* [21] uniquely alter stress-induced transcriptional kinetics, rendering borrowed internal controls highly erratic [20]. And the recent advent of full-length transcriptomics offers a powerful, unbiased methodology for capturing a comprehensive, assembly-free landscape of all expressed isoforms [22]. Unlike traditional protocols that require the empirical testing of 15 or more generic housekeeping genes (many of which fail validation), transcriptome-wide datasets enable rigorous in silico pre-screening of the entire expression. In this study, classical universal reference genes such as *GAPDH*, *18S rRNA*, and *EF1α* were preemptively excluded from the RT-qPCR candidate pool due to their raw transcriptomic profiles exhibiting statistically significant volatility under the applied stresses. Consequently, the nine selected candidate genes, comprising specific, highly stable paralogs from massively expanded gene families (e.g., *Actin-101* and *HSP70-1*), represent a highly distilled, pre-validated cohort. This approach enables isoform-level resolution and reduces the need for extensive empirical screening. It facilitates the targeted discovery of novel, non-traditional reference genes that maintain transcriptional stability across diverse stress libraries [23].

This study presents the first validated RT-qPCR standardization framework for *P. zhennan*. By utilizing a calibrated full-length transcriptome database, nine highly stable candidate reference genes were selected, and their expression stability was systematically evaluated across abiotic (drought) and biotic (*C. fructicola* disease) stresses. Through the comprehensive integration of four distinct statistical algorithms (Delta Ct, geNorm, NormFinder, and BestKeeper) alongside the RefFinder consensus tool, the analysis successfully identified optimal condition-specific reference genes (*CYP20-1*/*HSP70-1* for drought; *Actin-101*/*Actin* for disease) as well as a robust universal combination (*Actin-101* and *β-Tubulin*) for cross-condition normalization. The reliability of these identified internal controls was conclusively validated through the empirical quantification of known stress-responsive transcripts, confirming a highly significant correlation with corresponding RNA-seq data. Ultimately, these results identify suitable reference genes that can accurately normalize gene expression in *P. zhennan*. This providing a vital reference for future molecular studies on this species and a methodological example for other non-model timber species.

## 2. Results

### 2.1. Primer Specificity and Amplification Efficiency of Candidate Reference Genes

**Figure 1:**
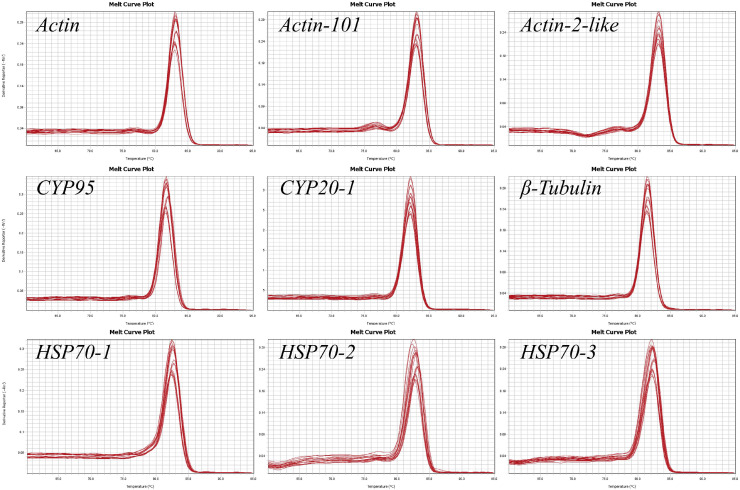
Primer specificity of the candidate reference genes. qPCR melt curve analyses of the nine candidate reference genes (*Actin*, *Actin-101*, *Actin-2-like*, *CYP95*, *CYP20-1*, *β-Tubulin*, *HSP70-1*, *HSP70-2*, and *HSP70-3*). All primer pairs exhibit a single, sharp melting peak, confirming the complete absence of non-specific amplification and primer-dimers.

To establish a robust standardization framework for gene expression analysis, we selected nine candidate reference genes and evaluated their primer specificity and amplification efficiency using PCR and qPCR assays. Gel electrophoresis of the PCR amplicons confirmed the presence of specific fragments at the expected sizes (Figure S1). Additionally, qPCR melt curve analyses across all *Phoebe zhennan* tissue cDNA templates yielded distinct, solitary peaks, confirming the absence of non-specific amplification or primer-dimers (Figure 1). Furthermore, standard curve analysis showed robust amplification efficiencies ranging from 96.8% to 105.9%, with strong linear correlations (*R*^2^ = 0.9943–0.9991) (Table S1). These findings indicate that the designed primer pairs exhibit high specificity and optimal efficiency required for RT-qPCR normalization, making them suitable for downstream quantitative studies.

### 2.2. Expression Profiles and Abundance of 9 Candidate Reference Genes

**Figure 2:**
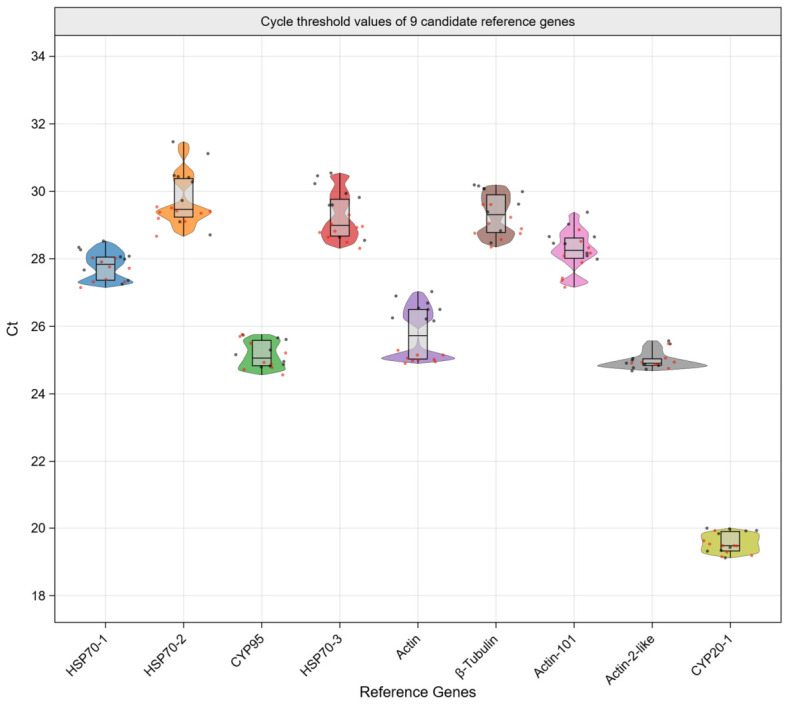
Distribution of cycle threshold (Ct) values for the nine candidate reference genes across all test samples. Violin plots combined with box plots illustrate the transcript abundance and expression variation of each gene in all *P. zhennan* samples. The horizontal black line within each box represents the median, while the upper and lower hinges correspond to the 75th and 25th percentiles, respectively. The violin shape indicates the probability density of the data, and the overlaid dots represent the actual Ct values of individual samples. Lower Ct values indicate higher transcript abundance.

To initially evaluate the transcript abundance and expression variation of the candidate reference genes, we analyzed the cycle threshold (Ct) values across all *P. zhennan* test samples (Figure 2). The results showed that the global Ct values ranged from 19.12 to 31.47. Notably, *CYP20-1* exhibited the lowest Ct values (ranging from 19.12 to 20.00), indicating the highest transcript abundance, whereas *HSP70-2* recorded the highest Ct values (28.67–31.47), reflecting the lowest expression level. Furthermore, the data distribution showed that *Actin-2-like* possessed the narrowest variation of less than one cycle (24.68–25.57), signifying virtually unchanged biological transcript abundance [24]. Closely followed by *CYP95* (24.56–25.76). Conversely, genes such as *HSP70-3* (28.31–30.54) and *Actin* (24.90–27.03) exhibited wider expression fluctuations, exceeding two cycles across the samples. These specific numerical findings indicate distinct differences in both expression levels and stability among the candidate genes. This underscores the necessity for further systematic statistical evaluation to identify the most reliable reference genes.

### 2.3. Comprehensive Evaluation and Validation of Optimal Reference Genes

To establish a robust normalization framework for Phoebe zhennan under diverse environmental stresses, we systematically evaluated nine candidate reference genes using four well-established statistical algorithms (ΔCt, geNorm, NormFinder, and BestKeeper). To address discrepancies among these methods, we then applied the comprehensive RefFinder tool to generate a consensus ranking.

#### 2.3.1. Delta Ct Analysis

**Figure 3:**
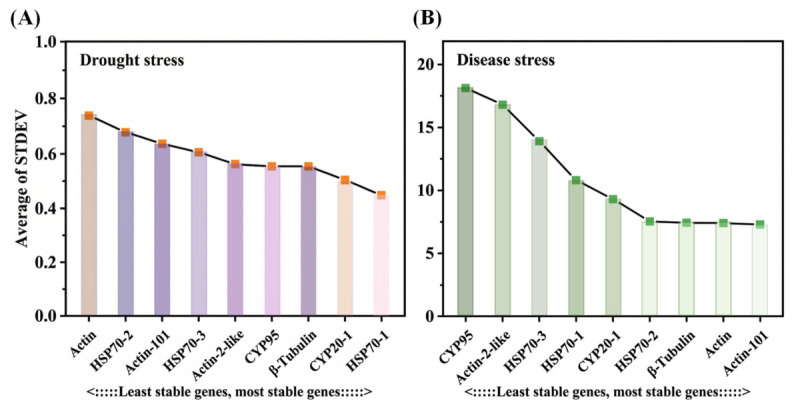
Expression stability analysis of the nine candidate reference genes based on the delta Ct method. The graphs present the average standard deviation (STDEV) of the candidate genes under (**A**) drought stress and (**B**) disease stress. The x-axis represents the stability ranking of the genes, with the arrow indicating the direction from the least stable to the most stable genes. A lower STDEV value indicates higher expression stability of the gene.

To determine the relative expression stability of the nine candidate reference genes under various stress conditions, we applied the delta Ct method to analyze the average standard deviation (STDEV) of gene expression fluctuations across the *P. zhennan* samples. This analysis revealed distinct stability rankings that were heavily dependent on the specific stress treatment applied. Under drought stress (Figure 3A), *HSP70-1* and *CYP20-1* exhibited the lowest average STDEV values, indicating they are the most stably expressed genes, while *Actin* was the least stable. In contrast, under disease stress (Figure 3B), *Actin-101* and *Actin* showed the lowest STDEV values, ranking them as the most stable genes, with *CYP95* identified as the least stable candidate. These findings highlight that reference gene stability in *P. zhennan* varies significantly under different stress conditions, necessitating careful selection for accurate quantitative analyses. The stability of reference genes in *P. zhennan* is highly condition-specific. This highlights the critical necessity to validate and tailor reference gene selection for specific experimental treatments before conducting downstream quantitative analyses.

#### 2.3.2. GeNorm Analysis

**Figure 4:**
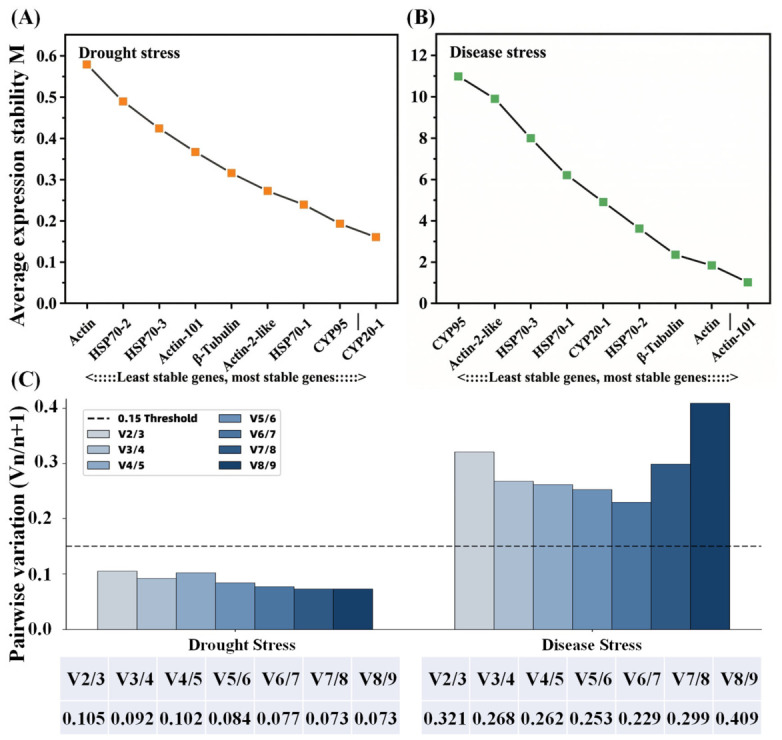
Pairwise variation (*V_n/n_*_+1_) analysis of the candidate reference genes calculated by geNorm. (**A**,**B**) Average expression stability (*M* values) determined by geNorm. Both (**A**,**B**) share the same Y-axis, representing the geNorm *M* value stability metric. A lower *M* value indicates greater stability. The X-axis shows the stepwise ranking of genes from the least to the most stable. (**C**) The pairwise variation values used to determine the optimal number of reference genes required for accurate normalization under drought and disease stresses. The dashed black line indicates the proposed geNorm threshold value of 0.15. A calculated value of *V_n/n_*_+1_ < 0.15 indicates that the inclusion of an additional (*n* + 1) reference gene is not required for reliable data standardization.

To evaluate the expression stability of the nine candidate reference genes under drought and disease stresses, we determined their mean stabilities by calculating the M values using geNorm. A lower value indicates greater stability, and significant differences were observed among the reference genes across the various stresses (Figure 4A,B). Specifically, as shown in Figure 4A, *CYP95* and *CYP20-1* emerged as the most stable genes under drought stress, whereas *Actin* was the least stable; conversely, *HSP70-1* and *Actin-101* displayed the highest stability under disease stress, while *CYP95* proved to be the least stable (Figure 4B). These results indicate that reference gene stability is highly specific to the conditions tested. This underscores the necessity of validating optimal internal controls for different experimental stress treatments.

Recognizing that a single reference gene usually does not meet the stability requirements for standardization, and that two or more reference genes are needed to reduce errors and obtain more accurate quantification of target gene expression, we utilized geNorm to calculate pairwise changes (*V_n/n_*_+1_) to determine the optimal number of reference genes for each stress. Using the established threshold of 0.15, our analysis revealed that all the *V_n/n_*_+1_ values fell below the cutoff under drought stress (Figure 4C). Because the raw expression profiles of *CYP20-1* and *Actin-2-like* exhibited minimal independent variance under drought stress, the step-wise inclusion of a third reference gene did not significantly alter the geometric mean. Consequently, the calculated pairwise variation (*V*_2/3_) remained well below the critical 0.15 threshold, confirming that a two-gene normalization strategy is statistically optimal and sufficient. Thus, the mathematically derived *V*_2/3_ value for this drought matrix will fall significantly below the mandated 0.15 threshold limit. The geNorm analysis provides a clear directive: for this specific abiotic drought stress analysis, the use of two optimal reference genes, specifically *CYP20-1* and *Actin-2-like*, is statistically sufficient for robust data normalization. Including a third gene does not enhance analytical resolution and would only consume additional biochemical resources.

The disease stress values reveal a chaotic and highly fractured transcriptional landscape, indicating that severe toll of pathogen invasion on host cellular machinery. Figure 4C dictates that for the erratic and highly degradative disease stress dataset, disease stress induced substantially greater gene variability, causing all values from *V*_2/3_ to *V*_8/9_ to exceed the standard 0.15 cutoff (reaching a minimum of *V*_6/7_ = 0.229). These findings confirm that utilizing just the two most stable candidates provides sufficient and accurate normalization for drought stress, but the strong stress-induced expression fluctuations under pathological conditions necessitate the identification of far more robust internal controls.

#### 2.3.3. NormFinder Analysis

**Figure 5:**
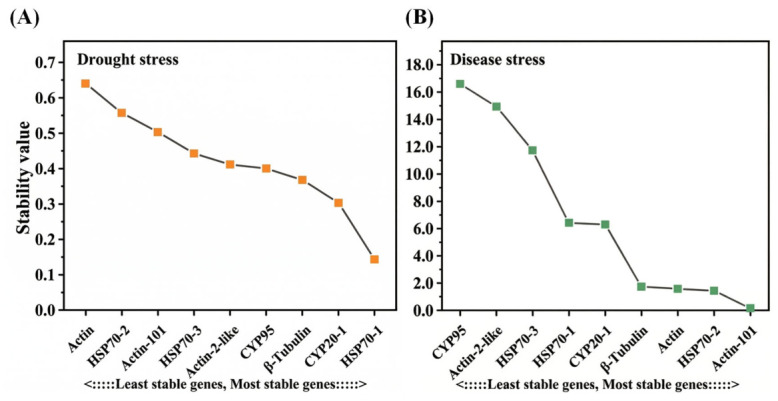
Expression stability analysis of the candidate reference genes using NormFinder software. The line charts present the stability values of the candidate genes under (**A**) drought stress and (**B**) disease stress. The x-axis represents the stability ranking of the genes, with the arrow indicating the direction from the least stable to the most stable genes. A lower stability value indicates higher expression stability.

To further validate the expression stability of the candidate reference genes, we analyzed the dataset using NormFinder (Version 0.953) software, where a lower stability value indicates higher expression stability (Figure 5, Table 1). The analysis revealed significant variation in the stability rankings of the candidate genes based on the applied stress. Under drought stress, *HSP70-1* (0.142) and *CYP20-1* (0.304) exhibited the lowest stability values, identifying them as the most stable genes, whereas *Actin* (0.639) ranked as the least stable. The results were highly consistent with those of the Delta CT analysis (identifying *HSP70-1* and *CYP20-1* as the most stable), while geNorm also placed *CYP20-1* in the top stability tier (tied with *CYP95*). Notably, Delta CT, geNorm, and NormFinder consistently showed that *Actin* and *HSP70-2* exhibited poor stability under drought stress in *P. zhennan*. Under disease stress, *Actin-101* (0.147) and *HSP70-2* (1.470) were identified as the most stably expressed genes, aligning closely with the Delta CT results. In contrast, *CYP95* (16.655) was ranked as the least stable candidate. All three algorithms consistently indicated that *Actin-2-like* and *CYP95* exhibited the poorest stability. In the comprehensive evaluation of all samples (Total), *Actin-101* (0.215) and *β-Tubulin* (0.800) ranked as the optimal reference genes. These results suggest that *β-Tubulin* and *Actin-101* form the best universal reference gene combination for accurate normalization across various stress conditions.

#### 2.3.4. BestKeeper Analysis

BestKeeper ranks candidate reference genes based on the standard deviation (SD) and coefficient of variation (CV) of the amplicon Cq variation in the RT-qPCR analysis. The most stable genes showed the lowest SD ± CV values, with SD values also below 1. To further evaluate the expression stability of the 9 candidate reference genes under drought and disease stress, we utilized the BestKeeper algorithm (Table 2). The analysis revealed that all candidate genes under drought stress had SD values of less than 1. *Actin-2-like* and *CYP20-1* exhibited the lowest SD and CV values (SD ± CV = 0.19 ± 0.78% and 0.26 ± 1.32%, respectively), indicating optimal expression stability. In contrast, *Actin* (0.74 ± 2.86%) and *HSP70-2* (0.64 ± 2.15%) displayed the highest statistical variations. These results suggest that *Actin-2-like* and *CYP20-1* are the most stably expressed candidates under drought stress according to BestKeeper parameters, whereas *Actin* shows excessive fluctuation, making it unsuitable for accurate normalization. Under disease stress, *Actin-101* exhibited the lowest SD and CV values (SD ± CV = 0.17 ± 0.58%), followed closely by *β-Tubulin* (0.82 ± 2.53%) and *Actin* (0.83 ± 2.48%). These three candidates met the critical inclusion criterion of an SD value less than 1. In contrast, other genes yielded SD values greater than 1, indicating substantial overall expression variation under disease conditions. And in the comprehensive evaluation encompassing all samples (total), *Actin-101* still yielded the lowest SD value (0.55), distinguishing itself as the sole candidate gene with an SD below the critical threshold of 1, followed by *β-Tubulin* (1.57) and *HSP70-2* (2.59). In contrast, *Actin-2-like* (8.52) and *CYP95* (8.02) displayed the highest SD values, reflecting maximum transcriptomic variability. Collectively, these results indicate that *Actin-101* possesses the most robust universal expression stability across diverse stress conditions.

### 2.4. Comprehensive Stability Analysis of Reference Genes Using RefFinder

**Figure 6:**
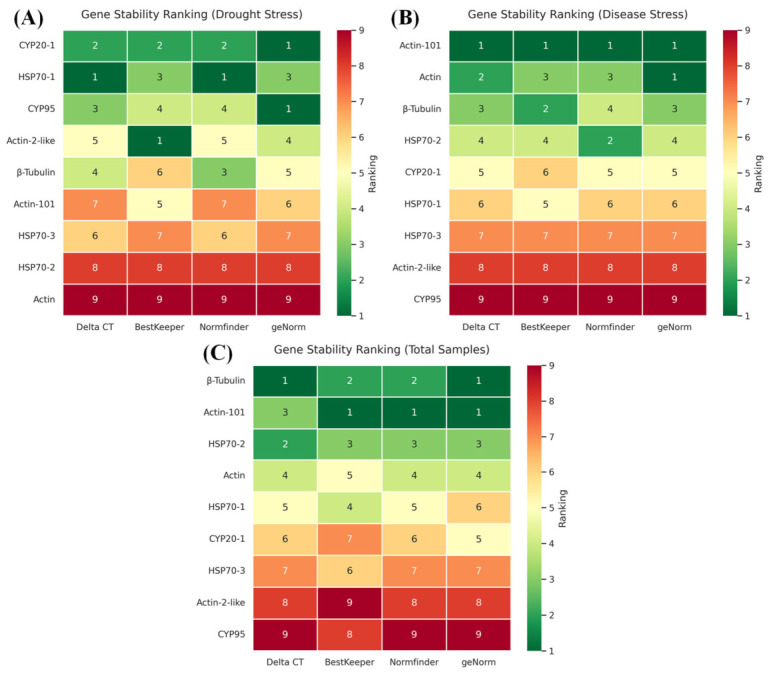
Heatmap of expression stability rankings for candidate reference genes under different experimental conditions. (**A**) Stability rankings under drought stress. (**B**) Stability rankings under disease stress. (**C**) Comprehensive stability rankings across all experimental samples (Total). The stability of nine candidate genes was evaluated using four different algorithms: Delta CT, BestKeeper, Normfinder, and geNorm. The color gradient from dark green to dark red corresponds to the ranking order from 1 (most stable) to 9 (least stable). Numbers within the cells denote the specific rank assigned by each corresponding algorithm.

As individual algorithms inherently vary and can yield discrepant stability assessments, relying on a single analytical method may introduce bias. To reduce the influence of the limitations and deviations of a single algorithm, a comprehensive analysis of four programs (∆Ct, geNorm, NormFinder, and BestKeeper) was conducted to identify the most suitable reference genes. As shown in Figure 6, the heatmaps clearly illustrate that *β-Tubulin* and *Actin-101* consistently emerged as the two most stable genes across ∆Ct, NormFinder, and BestKeeper methodologies, with geNorm identifying them as co-ranked optimal candidates. Furthermore, *HSP70-2* consistently ranked among the top three candidates. In stark contrast, all four algorithms unanimously identified *Actin-2-like* and *CYP95* as the least stable candidates, consistently placing them in the bottom two positions. To further mitigate potential biases and overcome the limitations inherent to individual algorithms, the comprehensive tool RefFinder was used to establish a definitive consensus ranking for the nine candidate reference genes (Table 3). Under drought stress, the RefFinder analysis identified *CYP20-1* and *HSP70-1* as the most stably expressed genes, while *Actin* ranked as the least stable candidate. Conversely, under disease stress, *Actin-101* and *Actin* emerged as the optimal reference genes, with CYP95 exhibiting the lowest stability. Notably, when evaluating the merged dataset across all experimental conditions, *Actin-101* and *β-Tubulin* secured the top two positions for stability, whereas *Actin-2-like* and *CYP95* were confirmed as the most unstable. These comprehensive findings indicate that *CYP20-1* and *HSP70-1* are ideal for drought experiments, while *Actin-101* and *Actin* are suitable for disease assays. Furthermore, the combination of *Actin-101* and *β-Tubulin* represents the most robust universal reference pair for accurate cross-condition normalization.

### 2.5. Validation of the Selected Reference Genes via Correlation Analysis of RNA-Seq and RT-qPCR Data

**Figure 7:**
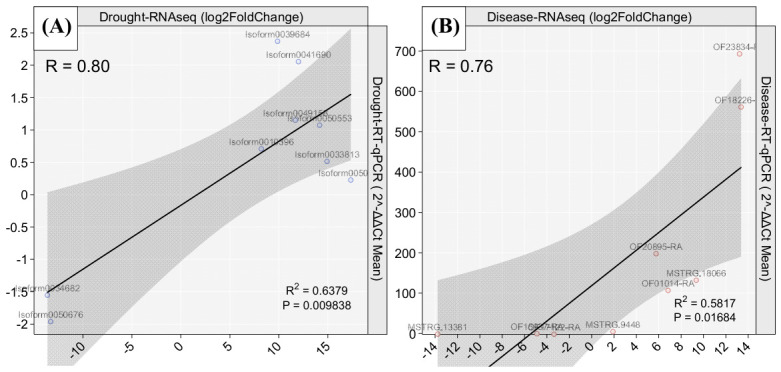
Correlation analysis of gene expression levels obtained from RNA-seq and RT-qPCR. The linear regression between the RNA-seq log2 fold change (x-axis) and the RT-qPCR relative expression levels (2^−ΔΔCt^, y-axis) for selected stress-responsive genes under (**A**) drought stress and (**B**) disease stress (*Colletotrichum fructicola* infection). The RT-qPCR data were normalized using the optimal reference gene pairs identified for each specific condition (*CYP20-1* and *HSP70-1* for drought; *Actin-101* and *Actin* for disease). The correlation coefficient (*R*), coefficient of determination (*R^2^*), and *p*-value are indicated in each panel. The grey shaded areas represent the 95% confidence intervals of the regression lines.

To validate the reliability of the identified optimal reference genes, we quantified the expression profiles of previously characterized stress-responsive transcripts in *Phoebe zhennan* via RT-qPCR, utilizing *CYP20-1* and *HSP70-1* for drought normalization, and *Actin-101* and *Actin* for disease (*Colletotrichum fructicola* infection) normalization. Linear regression analysis (Figure 7) revealed a significant positive correlation between the RT-qPCR relative expression values (2^−ΔΔCt^) and the corresponding RNA-seq log2 fold changes for both the drought (*R* = 0.80, *p* = 0.0098; Figure 7A) and disease (*R* = 0.76, *p* = 0.0168; Figure 7B) datasets. This concordance directly cross-validates our transcriptomic data and demonstrates the accuracy and reproducibility of these reference genes for normalizing gene expression in *P. zhennan* under diverse biotic and abiotic stresses.

To further substantiate the necessity of this framework, a rigorous negative control analysis was performed by normalizing the identical stress-responsive target transcripts against lower-ranked candidate genes (Table 4). The analysis clearly demonstrates that normalization with unstable reference genes, specifically *Actin* under drought stress (*R*^2^ = 0.0142, *p* = 0.7618) and *CYP95* under disease stress (*R*^2^ = 0.0084, *p* = 0.8155), led to statistical distortion. These results further support the importance of selecting condition-specific reference genes for RT-qPCR normalization.

## 3. Discussion

In this study, we established the first validated RT-qPCR normalization framework for the economically and ecologically vital endangered timber species *Phoebe zhennan*. By leveraging the full-length transcriptomic data combined with a multi-algorithm statistical approach, we demonstrated that the expression stability of candidate reference genes in *P. zhennan* is highly dependent on the specific stress condition. This reinforces the established principle that reference genes must be validated for each species and experimental context.

For decades, as indispensable tools for quantifying gene expression in molecular biology, the use of reference genes has traditionally relied on the assumption that genes governing basal cellular metabolism, such as those encoding *Actins* and *Tubulins*, are static and universally conserved across plant species [25]. However, our transcriptomic data suggest that a universally stable “housekeeping” gene is unlikely to exist across non-model woody plants. The use of unvalidated, historically accepted reference genes can distort transcriptional data, potentially leading to inaccurate biological interpretations [26]. This limitation is increasingly recognized in plant genomics literature, which cautions against the direct extrapolation of reference genes from herbaceous model species like *Arabidopsis thaliana* to complex woody plants [27].

Our findings align with the updated Minimum Information for Publication of Quantitative Real-Time PCR Experiments (MIQE 2.0) guidelines, which mandate the empirical validation of condition-specific reference genes. When *P. zhennan* seedlings were subjected to physiological stress, the expression of several traditional metabolic genes fluctuated significantly. For example, geNorm and NormFinder analyses revealed that commonly accepted reference genes, such as *Actin*, were highly unstable during drought stress, while *CYP95* exhibited high expression variation during fungal pathogenesis. This degree of transcriptional variation under environmental stress is not unique to *P. zhennan*; similar phenomena have been documented in other plant species. In sword-leaf dogbane (*Apocynum venetum*), the expression of commonly used housekeeping genes varied significantly based on whether the plant was subjected to salinity or biological elicitors [28]. In papaya (*Carica papaya*), classical reference genes such as *Actin*, *18S rRNA*, and *GAPDH* were found to be entirely unsuitable across various developmental stages and storage temperatures, necessitating the validation of alternative factors like *EIF* and *TBP1* [29]. By utilizing unbiased, assembly-free full-length isoform sequencing to identify novel, condition-specific references, our framework addresses a critical analytical challenge. Full-length transcriptomics allows for the precise screening of specific transcriptional isoforms, minimizing the analytical noise that occurs when traditional RNA-seq or microarray platforms quantify multiple, differentially regulated paralogs within large gene families [30]. Our methodology therefore provides a robust framework for future molecular investigations, ensuring that the quantification of stress-responsive gene expression in *P. zhennan* rests upon a solid statistical foundation.

Our first major conclusion is that *HSP70-1* and *CYP20-1* are stable under abiotic drought stress. Our multi-algorithm analysis utilizing the Delta Ct method, NormFinder, geNorm, and BestKeeper unanimously identified the specific *HSP70-1* isoform as one of the most stable reference genes for *P. zhennan* under prolonged drought stress. Under the BestKeeper algorithm, which evaluates stability based on the standard deviation (SD) and coefficient of variation (CV) of raw Cq values, *HSP70-1* consistently maintained an SD well below the critical threshold of 1.0, indicating minimal expression variance. This observation suggests potential functional divergence within the *HSP70* gene family in the Lauraceae lineage. The *P. zhennan* genome likely contains numerous of *HSP70* paralogs, much like the 72 *CsHSP70* genes identified in *Camelina sativa* [31] or the 28 *BvHSP70* genes in sugar beet [32]. While our results showed that other isoforms, such as *HSP70-2* and *HSP70-3*, exhibited high statistical variation, reflecting their standard stress inducibility. However, the specific HSP70-1 transcript maintains consistent expression under drought stress, likely reflecting functional divergence within the expanded HSP70 family where certain paralogs retain constitutive roles rather than stress-inducible functions. We hypothesize that this specific isoform performs basal constitutive chaperone duties that remain constant to sustain basic cellular viability during dehydration. The capacity to differentiate this stable constitutive paralog from its variable, stress-responsive sister genes highlights the high resolution of full-length transcriptomics in selecting reference genes. Equally notable is the identification of *CYP20-1* as a co-optimal reference gene under drought stress. Cyclophilins (CYPs) are ubiquitous peptidyl-prolyl cis-trans isomerases that catalyze the folding of proteins and have been frequently implicated in stress adaptation, particularly in adjusting photosynthetic complexes during drought-induced photoinhibition and oxidative stress. While cyclophilins can be stress-inducible in some contexts, our finding that *CYP20-1* maintains high transcriptional stability in *P. zhennan* aligns with studies in other species. For instance, in *Glycyrrhiza uralensis* (licorice), *CYP* was identified as one of the most stable reference genes in leaves under salt stress [33]. Similarly, a comprehensive evaluation in maize (*Zea mays*) across various abiotic stresses, phytohormone treatments, and tissue types confirmed *CYP* as one of the most reliable reference genes for overall gene expression normalization [34]. Consistent findings have also been reported in *Apocynum venetum* [35]. The geNorm pairwise variation analysis (*V*_*n*/*n*+1_) provided statistical support for this combination: the calculated *V*_2/3_ value for the drought matrix fell well below the recommended 0.15 threshold, indicating that the paired utilization of *CYP20-1* and *HSP70-1* (or *Actin-2-like*, which also showed high stability under drought) is sufficient to achieve robust data normalization.

Our second key conclusion focuses on the transcriptional dynamics of *P. zhennan* during biotic stress, wherein we identified *Actin-101* and *Actin* as the optimal reference genes for transcriptomic responses during *Colletotrichum fructicola* infection. *C. fructicola* is a highly aggressive hemibiotrophic fungal pathogen responsible for significant outbreaks of anthracnose and twig blight in woody plants [19]. The observation that the specific *Actin-101* isoform maintains stable expression in *P. zhennan* during both the active colonization and subsequent necrotrophic tissue destruction by *C. fructicola* suggests a potential evolutionary specialization of the host’s *Actin* gene family. While certain vegetative *Actin* isoforms undergo rapid transcriptional reprogramming to facilitate the localized immune vesicle trafficking required for defense [36] and differentially regulated during plant-pathogen interactions [37]. The stability of *Actin-101* is supported by our BestKeeper analysis, which produced a low standard deviation (SD = 0.17) and coefficient of variation (CV = 0.58%). Likewise, in *C. gloeosporioides* interacting with guava (*Psidium guajava*), the tubulin gene (*CgTUB2*) was shown to be highly stable [38].

The present study still contains inherent experimental limitations that leave several broader ecological, physiological, and genetic questions regarding *P. zhennan* temporarily unanswered. Primarily, our study validated reference genes solely under isolated drought and disease conditions. In its natural habitat, however, *P. zhennan* faces complex combinatorial stresses, including the simultaneous occurrence of drought and disease pressures due to habitat fragmentation [18,39]. Since extreme heat triggers extensive transcriptomic reorganizations, rapid RNA decay, and alternate chaperone network upregulation [40,41], *CYP20-1*, *HSP70-1*, and *Actin-101* may exhibit unacceptable volatility under such untested conditions. Similarly, although high salinity and waterlogging are not considered primary mortality drivers within the species’ native subtropical mountain habitat, these environmental factors would still require independent reference gene validation if *P. zhennan* were cultivated under ex situ conditions. The transcriptional stability of *CYP20-1*, *HSP70-1*, and *Actin-101* has yet to be empirically evaluated under complex environmental variables, including extreme thermal fluctuations, severe UV radiation, and systemic salt exposure [42,43]. In addition, whether these candidate reference genes maintain stable expression during physiological recovery or tissue repair processes remains unclear.

Furthermore, our study’s reliance on *P. zhennan* seedlings restricts our transcriptomic perspective to the juvenile stage. While prioritizing seedlings is conservationally logical given their high vulnerability to drought and anthracnose mortality [19,44,45], *P. zhennan* is a slow-growing perennial species that requires several decades to develop its highly lignified, secondary metabolite-rich heartwood through intricate shifts in the phenylpropanoid and lignin biosynthetic pathways [18,20,21]. The epigenetic landscape and transcriptomic dynamics of a mature tree undergoing secondary xylem differentiation differ vastly from those of a vegetative seedling [46,47], leaving it uncertain whether *Actin-101* and *β-Tubulin* transcription rates shift fundamentally during decades of wood formation. Aging in trees involves distinct DNA methylation and chromatin remodeling [48]; thus, without longitudinal validation across the tree’s lifespan, applying these reference genes to mature timber or reproductive studies requires strict caution. While our framework provides a robust juvenile stress model, extrapolating these findings to broader climatic stressors or mature developmental stages requires further empirical validation.

## 4. Materials and Methods

### 4.1. Plant Materials and Experimental Design

One-year-old *Phoebe zhennan* seedlings, sourced from a standardized provenance in Laifeng County, Hubei Province, China, were maintained under controlled environmental conditions (uniform light, moisture, and nutrients) for one year. Seedlings exhibiting median growth parameters (height and basal diameter) were selected as representative experimental subjects. To assess reference gene stability under abiotic stress, tissue samples were collected from plants subjected to a drought treatment at 0 days (control) and 16 days. For biotic stress profiling, naturally diseased *P. zhennan* infected with the pathogen *Colletotrichum fructicola* and age-matched healthy control plants were harvested from their natural habitat. All collected tissues (comprising roots, stems, leaves, and buds) were immediately snap-frozen in liquid nitrogen and stored at −80 °C until RNA extraction.

While natural field infections were utilized for initial in silico transcriptomic screening to capture field-realistic physiological states, rigorous standardized artificial inoculations were performed to empirically validate the stability of the candidate reference genes. The hemibiotrophic pathogen was isolated from naturally diseased *P. zhennan* tissues, purified, and cultured on Potato Dextrose Agar (PDA).

Pathogenicity assays were strictly conducted on intact, whole plants rather than detached tissues to preserve systemic physiological responses. To account for age-dependent transcriptomic variations, inoculations were performed across a developmental gradient: 3-month-old seedlings, 2-year-old seedlings (assessing both emerging branches and tender stem sections), and 7-year-old saplings. To prevent cross-contamination along the monopodial main stem characteristic of juvenile *P. zhennan*, inoculations were strictly limited to a single site per plant. The experimental design was highly structured, utilizing three independent biological replicates, with five distinct plants per treatment per replicate (totaling 15 plants per condition). Across all age groups and tissue types, a total of 120 individual plants were utilized to ensure statistical robustness. Pathogenicity was conclusively confirmed via symptom expression and the fulfillment of Koch’s postulates.

### 4.2. RNA Isolation and cDNA Synthesis

Total RNA was extracted from 0.1 g of cryopreserved tissue using a plant-specific total RNA extraction kit (HiPure Plant RNA Plus Kit, Guangzhou Magen Biotechnology Co., Ltd., Guangzhou, China). RNA integrity and spectrophotometric concentrations were validated through 1% agarose gel electrophoresis and a Micro Drop spectrophotometer (BIO-DL, Shanghai, China), respectively. Subsequently, 800 ng of high-quality total RNA was reverse-transcribed into cDNA using random hexamers (N6) and a reverse transcriptase master mix (Hifair^®^ II Enzyme Mix, Yeasen Biotechnology, Shanghai, China). The thermal cycling profile for cDNA synthesis was 25 °C for 5 min, 42 °C for 30 min, and an inactivation step at 85 °C for 5 min. The resulting cDNA libraries were diluted tenfold and stored at −20 °C for downstream quantitative applications.

### 4.3. Candidate Reference Gene Selection and Primer Verification

Candidate reference genes were systematically identified by mining a highly calibrated *P. zhennan* full-length transcriptome database encompassing 12,458 transcripts. To ensure robust transcriptional stability across all sequenced stress libraries, rigorous bioinformatic filtering criteria were applied: an absolute log2 fold change (|log_2_*FC*|) < 0.5 and an adjusted *p*-value (padj) > 0.05. Furthermore, to guarantee reliable RT-qPCR detectability and mitigate stochastic sampling noise associated with low-abundance transcripts, a baseline expression threshold of mean FPKM > 10 was mandated across all libraries. This stringent filtering paradigm yielded a high-confidence subset of 12 stably expressed transcripts. Sequence annotations were confirmed via BLASTX alignments against the NCBI non-redundant (NR) database (https://blast.ncbi.nlm.nih.gov/Blast.cgi, accessed on 22 September 2025). Transcript-specific primers were designed targeting the coding sequences (CDS) using Primer Premier 5.0 software, adhering strictly to the following parameters: amplicon lengths of 100–200 bp, a theoretical primer melting temperature (*Tm*) window of 59–61 °C, and GC contents between 40–70%. Primer specificity was computationally validated utilizing the NCBI Primer-BLAST (http://www.ncbi.nlm.nih.gov/tools/primer-blast/, accessed on 23 September 2025) algorithm prior to commercial synthesis. Three transcripts were excluded due to suboptimal amplification efficiency or the presence of non-specific amplification peaks. Finally, nine specific genes were identified as elite candidate reference genes. All corresponding target-specific primer pairs (detailed comprehensively in Table 5) were synthesized by Qingdao STD Standard Testing CO., Ltd. (Qingdao, China). The comprehensive transcriptomic expression profiles (FPKM values) confirming the baseline stability of these final candidates are provided in Table S2.

### 4.4. Reverse Transcription Quantitative Real-Time PCR (RT-qPCR) Assays

The fragment of candidate reference genes was amplified using designed primers and a cDNA template from *P. zhennan* samples. The RT-qPCR assay was performed using the SYBR Green dye method. All RT-qPCRs were carried out by using the Hieff^®^ qPCR SYBR Green Master Mix (Low Rox Plus) (Yeasen Biotechnology Co., Ltd., Shanghai, China), and the reaction system (10 µL) was as follows: 5 µL of 1 × SYBR Green Master Mix; 2.5 µL of cDNA; 1 µL of each specific primer pair (2 μM forward and reverse primers); and RNase-free water was added to reach 10 μL. Additionally, a non-template control was included for each gene. RT-qPCR was conducted using the Applied Biosystems ViiA™ 7 Real-Time PCR system (ABI, Foster City, CA, USA) for amplification. To ensure synchronized and comparable amplification kinetics across all candidate genes within the same plate run, a unified thermal cycling profile was applied. The procedure was as follows: an initial pre-denaturation at 95 °C for 5 min, followed by denaturation at 95 °C for 10 s, and annealing and extension at a fixed temperature of 60 °C for 30 s, across 40 cycles. Amplicon specificity was verified through melting curve analyses, with continuous data acquisition from 60 °C to 95 °C, ensuring the absence of primer-dimers or off-target amplification. Assays were conducted using three biological replicates, each with three technical replicates. In addition, Cq values were used to draw standard curves, and the primer amplification efficiency (E = (10^[−1/slope]^ − 1) × 100%) and correlation coefficient (*R*^2^) were analyzed. The calculation of amplification efficiency requires that the standard curve has a satisfactory linear relationship (*R*^2^ > 0.99), and the amplification efficiency of all primers meeting the requirements of qPCR detection should be between 90% and 110%. The expression levels of candidate reference genes were measured by RT-qPCR analysis, using the GM-02 PCR system (Hangzhou Jingle Scientific Instrument Co., Ltd., Hangzhou, China).

### 4.5. Statistical Evaluation of Gene Stability

The baseline expression stability of the nine candidate genes was independently evaluated using four established statistical algorithms: Delta Ct, geNorm (v3.5), NormFinder (v0.953), and BestKeeper (v1.0). To comply with the algorithmic requirements of geNorm and NormFinder, raw cycle threshold (Ct) values were log-transformed into relative quantities (2^−ΔCt^). The geNorm algorithm was additionally utilized to calculate pairwise variation values (*V*_*n*/*n*+1_) to delineate the optimal number of internal controls necessary for accurate normalization, adopting the standard inclusion threshold of 0.15. To minimize the influence of limitations and deviations from a single algorithm, a comprehensive analysis was conducted using four programs (∆Ct, geNorm, NormFinder, and BestKeeper) to identify the most suitable reference genes. The RefFinder program was then used to integrate the discrete datasets and compute a consensus stability ranking. Final empirical validation of the optimal reference genes involved quantifying stress-responsive transcript levels using the 2^−ΔΔCt^ method. These RT-qPCR outputs were subsequently subjected to linear regression analysis against their corresponding RNA-seq log_2_ fold-change values to determine statistical correlation (*R* and *p* values).

### 4.6. Statistical Analyses

All quantitative data were obtained from a minimum of three independent biological replicates. Statistical significance was assessed using Student’s *t*-test for direct pairwise comparisons. To rigorously control Type I error rates during complex multi-gene analyses, Duncan’s New Multiple Range Test (DMRT) was employed to evaluate variance across three or more distinct experimental genes simultaneously. A significance threshold of *p* < 0.05 was applied across all tests. Data processing was conducted using Microsoft Excel (version 2023). For figure creation, Adobe Illustrator (version 2023), figdraw (version 2.0), and Microsoft PowerPoint (version 2023) were utilized.

## 5. Conclusions

The precise quantification of gene expression is fundamental to modern molecular biology. However, for complex, non-model woody plants, reliance on unvalidated, cross-species reference genes has historically led to inaccurate data interpretation. This study provides the first systematic identification and validation of optimal reference genes for the endangered and economically valuable timber species *P. zhennan* under both abiotic and biotic stress conditions. Utilizing Iso-Seq full-length transcriptomic data and a multi-algorithm statistical approach, we identified condition-specific reference genes for precise transcript normalization. Transitioning from high-volume empirical screening to targeted, transcriptome-guided pre-screening, researchers can significantly reduce experimental and biochemical waste. The reference genes identified here will facilitate accurate gene expression studies on drought and disease responses in *P. zhennan* seedlings. Moreover, the integrated methodology of full-length transcriptome screening and multi-algorithm validation offers a transferable strategy for developing similar standardization tools in other horticulturally and ecologically important plant species.

## 6. Patents

The work reported in this manuscript has resulted in a successfully registered computer software copyright: “RefGeneAutoScreener: An Automated Screening and Analysis System for Reference Genes” (Registration No. 2025SR1329674). The software automatically aggregates heterogeneous datasets and computes a comprehensive ranking based on the RefFinder geometric mean method, thereby enabling reproducible reference gene screening.

## Figures and Tables

**Table 1:** Expression stability values and rankings of candidate reference genes in *P. zhennan* under different stresses determined by NormFinder.

	Drought Stress		Disease Stress		Total	
Ranking	Gene	Stability	Gene	Stability	Gene	Stability
1	*HSP70-1*	0.142	*Actin-101*	0.147	*Actin-101*	0.215
2	*CYP20-1*	0.304	*HSP70-2*	1.470	*β-Tubulin*	0.800
3	*β-Tubulin*	0.368	*Actin*	1.592	*HSP70-2*	1.514
4	*CYP95*	0.401	*β-Tubulin*	1.744	*Actin*	2.927
5	*Actin-2-like*	0.413	*CYP20-1*	6.350	*HSP70-1*	4.404
6	*HSP70-3*	0.443	*HSP70-1*	6.467	*CYP20-1*	6.617
7	*Actin-101*	0.503	*HSP70-3*	11.721	*HSP70-3*	8.183
8	*HSP70-2*	0.554	*Actin-2-like*	14.953	*Actin-2-like*	11.543
9	*Actin*	0.639	*CYP95*	16.655	*CYP95*	11.902

**Table 2:** Expression stability of candidate reference genes in *P. zhennan* under different stress conditions analyzed by BestKeeper.

Ranking		1	2	3	4	5	6	7	8	9
Drought	Gene	*Actin-2-like*	*CYP20-1*	*HSP70-1*	*CYP95*	*Actin-101*	*β-Tubulin*	*HSP70-3*	*HSP70-2*	*Actin*
	SD	0.19	0.26	0.35	0.35	0.44	0.55	0.60	0.64	0.74
	CV [%]	0.78	1.32	1.26	1.40	1.57	1.86	2.06	2.15	2.86
Disease	Gene	*Actin-101*	*β-Tubulin*	*Actin*	*HSP70-2*	*HSP70-1*	*CYP20-1*	*HSP70-3*	*Actin-2-like*	*CYP95*
	SD	0.17	0.82	0.83	1.15	3.43	3.65	6.68	12.22	15.29
	CV [%]	0.58	2.53	2.48	3.29	0.58	11.79	12.22	17.78	16.67
Total	Gene	*Actin-101*	*β-Tubulin*	*HSP70-2*	*HSP70-1*	*Actin*	*HSP70-3*	*CYP20-1*	*CYP95*	*Actin-2-like*
	SD	0.55	1.57	2.59	3.18	3.80	3.85	5.68	8.02	8.52
	CV [%]	1.92	5.08	6.01	8.84	10.83	10.97	12.50	13.33	18.89

**Table 3:** Comprehensive stability ranking of the candidate reference genes in *P. zhennan* under different stress conditions as determined by RefFinder.

Ranking	Drought Stress		Disease Stress		Total	
	Gene	Values	Gene	Values	Gene	Values
1	*CYP20-1*	1.68	*Actin-101*	1	*Actin-101*	1.32
2	*HSP70-1*	1.73	*Actin*	2.06	*β-Tubulin*	1.41
3	*CYP95*	2.63	*β-Tubulin*	2.91	*HSP70-2*	2.71
4	*Actin-2-like*	3.16	*HSP70-2*	3.36	*Actin*	4.23
5	*β-Tubulin*	4.36	*CYP20-1*	5.23	*HSP70-1*	4.95
6	*Actin-101*	6.19	*HSP70-1*	5.73	*CYP20-1*	5.96
7	*HSP70-3*	6.48	*HSP70-3*	7.00	*HSP70-3*	6.74
8	*HSP70-2*	8.00	*Actin-2-like*	8.00	*Actin-2-like*	8.24
9	*Actin*	9.00	*CYP95*	9.00	*CYP95*	8.74

**Table 4:** Linear regression correlation coefficients (*R*^2^) and significance (*p*-values) comparing RT-qPCR and RNA-seq expression data.

Drought Stress			Disease Stress		
Reference Gene	*R* ^2^	*P*	Reference Gene	*R* ^2^	*P*
*HSP70-1*	0.6124	0.0125 *	*Actin*	0.5085	0.0315 *
*Actin-2-like*	0.5842	0.0161 *	*β-Tubulin*	0.4682	0.0416 *
*CYP95*	0.4981	0.0335 *	*HSP70-* *3*	0.4054	0.0224 *
*β-Tubulin*	0.4512	0.0471 *	*CYP20-1*	0.3088	0.1205
*Actin-101*	0.3804	0.0762	*HSP70-2*	0.2185	0.2052
*HSP70-3*	0.2541	0.1668	*HSP70-* *1*	0.1214	0.3588
*HSP70-* *2*	0.1495	0.4635	*Actin-2-like*	0.0521	0.5542
*Actin*	0.0788	0.7618	*CYP95*	0.0184	0.8155

* Indicates significant correlation at the 0.05 level.

**Table 5:** Candidate reference genes and primer sequences used in this study.

Gene Symbol	Putative Function	Gene No.	Gene Name	Primer Sequence (5′-3′)	Product (bp)
*ACT*	essential cytoskeletal structural components	Isoform0044008	*Actin-101*	CTCTCTATGCCAGTGGTCGT	130
				TCACGACCTGCAAGATCCAG	
		Isoform0034540	*Actin*	GGCCTACATTGCCCTTGACT	113
				GCTCCGCCCCAATGGTAATA	
		Isoform0048328	*Actin-2-like*	GGCCGTACCACAGGTATTGT	107
				GCAAGGTCAAGCCGGAGAAT	
*Beta-tubulin*	cytoskeletal component	Isoform0040625	*β-Tubulin*	TGGATCTGGGATGGGAACCT	126
				GCATTGTACGGCTCAACCAC	
*CYP*	peptidyl-prolyl cis-trans isomerases critical for protein folding	Isoform0050648	*CYP20-1*	TGGCAAATGCTGGTCCTGAT	170
				CACTTTCTGCTTGGGTGTGC	
		Isoform0025490	*CYP95*	GCGAATGCTGGTCCTGATAC	159
				AACAGGCTTTGCCTCGTCAG	
*HSP70*	constitutive ATP-dependent molecular chaperones	Isoform0001533	*HSP70-1*	AATTCAGGGCGGCATCCTTC	101
				TGGTGAAGATACCTCCTAGCG	
		Isoform0011934	*HSP70-2*	TTTGAGGTGAAGGCCACAGC	163
				TAGCCCTCTCACATGCAGTC	
		Isoform0032092	*HSP70-3*	TGGAGACACGCATCTTGGAG	149
				TCCTCTTAGCCCTCTCGCAT	

## Data Availability

The original contributions presented in this study are included in the article/Supplementary Materials. Further inquiries can be directed to the corresponding author.

## Supplementary Materials

The following supporting information can be downloaded at: https://www.mdpi.com/article/10.3390/plants15111736/s1, Figure S1: Agarose gel electrophoresis of total RNA extracts from samples; Table S1: Amplification efficiency of candidate reference gene primer pairs; Table S2: Transcriptomic expression profiles and stability metrics of the candidate reference genes in *Phoebe zhennan*.

## Abbreviations

The following abbreviations are used in this manuscript:

2^−ΔΔCt^	relative quantification method
ACT	actin
CDS	Coding sequences
Cq / Ct	Cycle threshold
CV	Coefficient of variation
CYP	cyclophilin
EF1α	Transcription elongation factor 1-alpha
HSP70	heat shock protein 70
MIQE	Minimum Information for Publication of Quantitative Real-Time PCR Experiments
NR	Non-redundant
*P*	probability value
PDA	Potato Dextrose Agar
RT-qPCR	Reverse transcription quantitative real-time PCR
*R* *^2^*	coefficient of determination
SD	Standard deviation
STDEV	Standard deviation
TUB	β-tubulin
ΔCt	delta cycle threshold
