# Supplementary material for: Comparative Analysis of Full-Length Reference Gene Stability in Phoebe zhennan Under Primary Abiotic and Biotic Stresses
Source: Plants (Basel). 2026 Jun 3;15(11):1736. doi: 10.3390/plants15111736 (PMC13259038; doi:10.3390/plants15111736)
Supplement: Supplementary file 1 [file plants-15-01736-s001.zip › plants-4286659-supplementary.pdf]

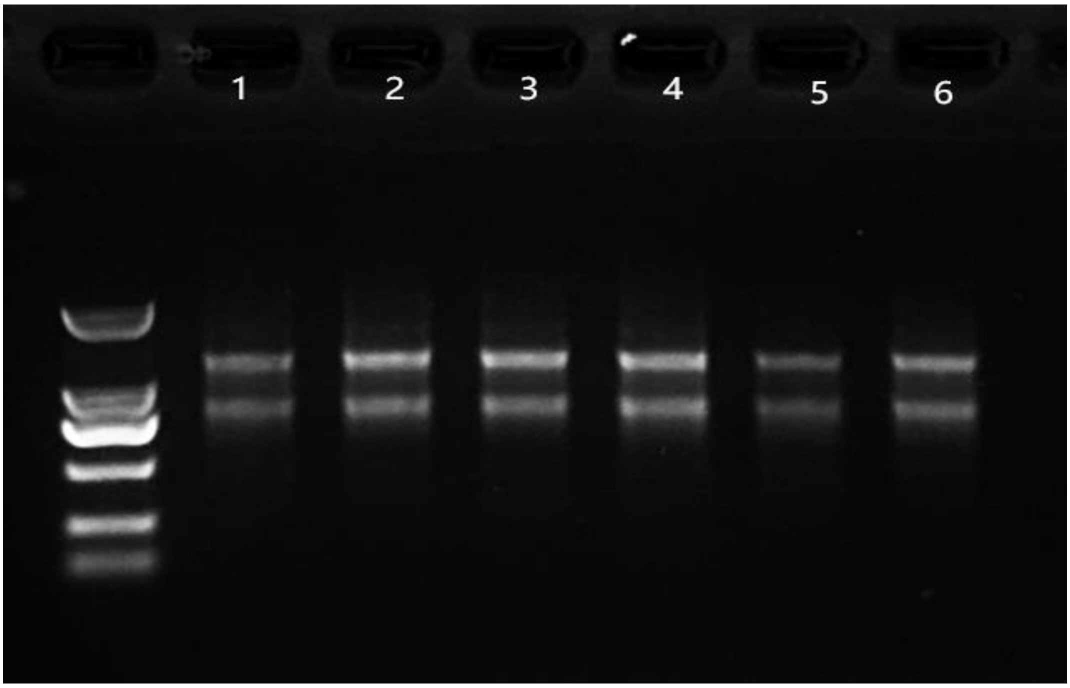

Figure S1. Agarose gel electrophoresis of total RNA extracts from samples. The results of a 1.5% agarose gel electrophoresis of six total RNA samples extracted from different treatments or tissues. Lanes 1–6 represent total RNA extracted from different samples. The distinct, sharp bands corresponding to the 28S and 18S ribosomal RNA (rRNA) in lanes 1–6, without noticeable degradation smearing, indicate good integrity of the total RNA, rendering it highly suitable for subsequent downstream applications.

Table S1. Amplification efficiency of candidate reference genes primer pairs.

Primer name	Slope	R^2	Amplification efficiency (%)
Actin-2-like	-3.3582	0.9982	98.5
Actin-101	-3.2980	0.9951	101.0
Actin	-3.4005	0.9975	96.8
β-Tubulin	-3.2201	0.9991	104.4
CYP20	-3.2753	0.9964	102.0
CYP95	-3.3321	0.9988	99.6
HSP70-1	-3.1891	0.9943	105.9
HSP70-2	-3.3812	0.9977	97.6
HSP70-3	-3.2504	0.9985	103.1

Table S2. Transcriptomic expression profiles and stability metrics of the candidate reference genes in *Phoebe zhennan*.

Gene Symbol	Transcript ID	Mean FPKM	$\log_2 FC$	lfcSE	stat	*padj*
CYP20-1	Isoform0050648	85.42	0.1807	0.0418	4.3185	0.4501
HSP70-1	Isoform0001533	120.35	-0.0253	0.1922	-0.1316	0.9211
CYP95	Isoform0025490	45.28	-0.1169	0.2742	-0.4265	0.7364
Actin-2-like	Isoform0048328	68.73	-0.3292	0.0365	-9.0083	0.1552
β-Tubulin	Isoform0040625	92.16	-0.4125	0.0423	-9.7524	0.3154
Actin-101	Isoform0044008	150.84	0.296	0.3445	0.8593	0.4727
HSP70-3	Isoform0032092	34.55	0.0851	0.2114	0.4025	0.6482
HSP70-2	Isoform0011934	28.41	0.3126	0.0451	6.9259	0.2198
Actin	Isoform0034540	105.67	-0.1333	0.5618	-0.2374	0.8561
